# Biometric analysis of the foetal meconium pattern using T1 weighted 2D gradient echo MRI

**DOI:** 10.1259/bjro.20200032

**Published:** 2020-08-05

**Authors:** Georgia Hyde, Andrew Fry, Ashok Raghavan, Elspeth Whitby

**Affiliations:** 1 Academic Unit of Reproductive and Developmental Medicine, The University of Sheffield, Jessop Wing, Tree Root Walk, Sheffield, S10 2SF, United Kingdom; 2 Medical Imaging and Medical Physics, Sheffield Teaching Hospitals NHS Trust, Royal Hallamshire Hospital, Beech Hill Road, S10 2JF, Sheffield, United Kingdom; 3 Department of Radiology, Sheffield Children’s Hospital, Clarkson Street, Sheffield S10 2TH, United Kingdom

## Abstract

**Objectives:**

Foetal MRI is used to assess abnormalities after ultrasonography. Bowel anomalies are a significant cause of neonatal morbidity, however there are little data concerning its normal appearance on antenatal MRI. This study aims to investigate the pattern of meconium accumulation throughout gestation using its hyperintense appearance on *T*_1_ weighted scans and add to the current published data.

**Methods:**

This was a retrospective cohort study in a tertiary referral clinical MRI centre. Foetal body MRI scans of varying gestational ages were obtained dating between October 2011 and March 2018. The bowel was visualised on *T*_1_ weighted images. The length of the meconium and the width of the meconium at the rectum, sigmoid colon, splenic flexure and hepatic flexure was measured. Presence or absence of meconium in the small bowel was noted. Inter- and intrarater reliability was assessed.

**Results:**

181 foetal body scans were reviewed. 52 were excluded and 129 analysed. Visualisation of the meconium in the large bowel became increasingly proximal with later gestations, and small bowel visualisation was greater at earlier gestations. There was statistically significant strong (*r* = 0.6–0.8) or very strong (*r* = 0.8–1.0) positive correlation of length and width with increasing gestation. Interrater reliability was moderate to excellent (*r* = 0.4–1.0).

**Conclusion:**

This study provides new information regarding the pattern of meconium accumulation throughout gestation. With care, the results can be used in clinical practice to aid diagnosis of bowel pathology.

**Advances in knowledge:**

The findings of this study provide further information concerning the normal accumulation of foetal meconium on MR imaging, an area where current research is limited.

## Introduction

Foetal structural abnormalities are primarily identified by ultrasound scan (USS) as part of routine antenatal care.^1^ Although cost-effective and widely used, screening ultrasonography is sometimes limited in the amount of information it can provide.^2^ In the last decade, MRI has become more widely available and is used to image a foetus after ultrasound, when the results are inconclusive or require further investigation.^3^

The majority of research in foetal MRI has thus far been concerned with neurological imaging.^4^ However, its use as a diagnostic technique for foetal body abnormalities is useful, especially in terms of using meconium to observe the anatomy of the bowel due to its hyperintense appearance on *T*_1_ weighted (*T*_1_W) imaging.^5^ Moreover, several studies have demonstrated the efficacy of foetal MRI in identifying and confirming conditions such as colonic obstruction and atresia.^6–9^ Even virtual colonography has been reported in foetal imaging.^10^

Before studying the use of MRI in foetuses with suspected bowel pathology, the normal appearance of the gatrointestinal (GI) tract must be considered. Current research regarding the normal appearance of the foetal bowel on MRI is limited. Particularly, there are little data describing the anatomical distribution of meconium throughout gestation in the large bowel, as the published small studies (less than 45 cases in each) analyses the data in two gestational age groups (less than 32 weeks and greater than 32 weeks).^7,8^ There are also no data for the small bowel, which does not appear to follow the same proximal accumulation throughout gestation as the large bowel.^8,9^ There are also a lack of data for gestations earlier than 20 weeks. This study aimed to assess the meconium pattern in the foetal bowel from 17 weeks gestation in a large cohort of confirmed normal gastrointestinal tract cases.

## Methods and materials

Foetal body scans from MR imaging of pregnant females between October 2011-May 2018 were reviewed. The females included in the study had been offered a MRI at a tertiary referral centre radiology department as part of routine clinical practice, after concerns from their ultrasound screening scan or a family history of previous abnormality. No additional imaging was required for the study. Scans were excluded from the study if the meconium could not be visualised for reasons such as insufficient scan quality or artefact, if bowel pathology was detected, if there were no T1 images or if a 3 T scanner was used. Follow-up data were obtained for all included cases using clinical data at birth. Local ethical approval was obtained from an ethics committee, and all methods were carried out in accordance with relevant guidelines and regulations.

MR images were obtained using a Siemens Avanto 1.5 T (Erlangen, Germany), and Siemens Magnetom Aera 1.5 T (Erlangen, Germany). A *T*_1_ W two-dimensional fast low angle shot (FLASH) sequence was used to image the foetal body in at least the coronal and sagittal planes as part of the routine protocol which included *T*_2_W and diffusion-weighted images. The T1 sequence acquisition parameters were as follows: time to repeat (TR)/time to echo (TE) 264/5 ms; field of view 288 mm; matrix 192 × 192; flip angle 70°; 20 slices of 4–5 mm thickness. All images were viewed with IMPAX (v. 6.5.3.3009, AGFA, Belgium) picture archiving and communication systems (PACS) software. Cases were identified from a master foetal MRI database. Gestational age and related pathology were noted for each scan.

**Figure 1.**
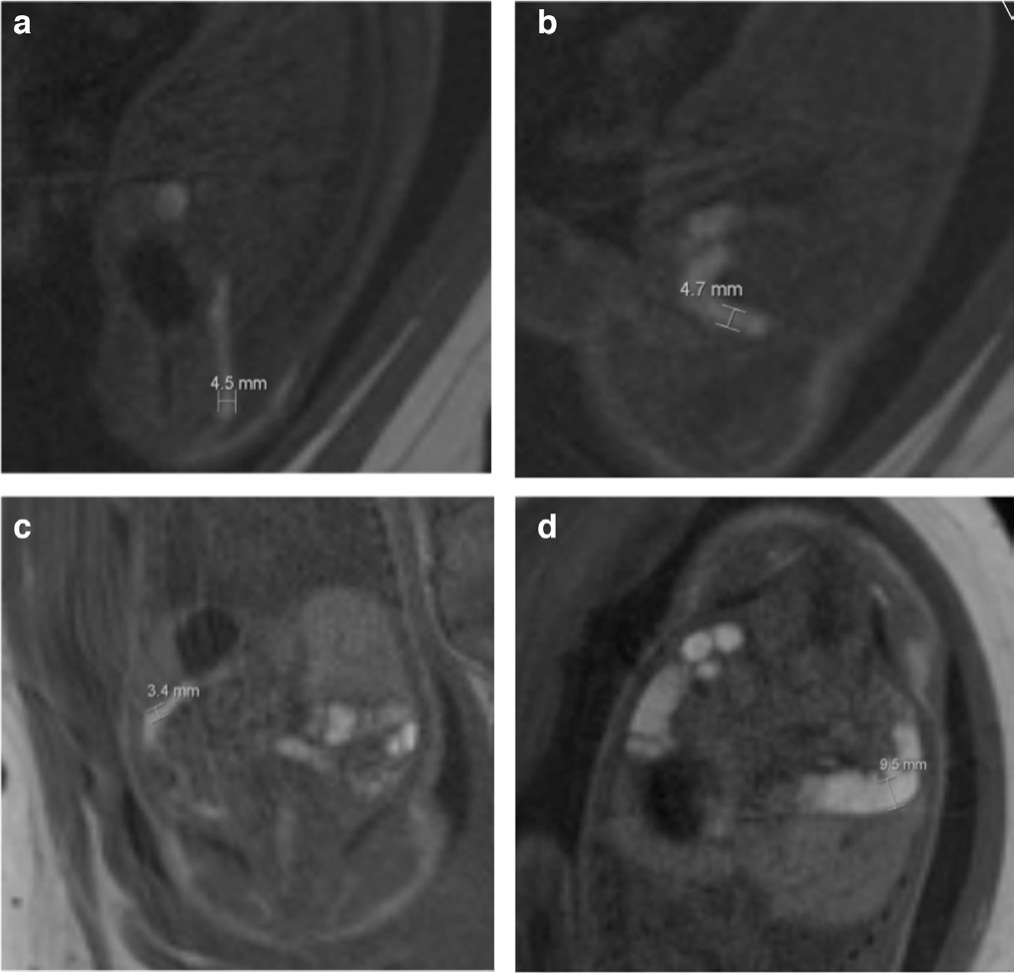
a-d: Bowel width measurements using the caliper tool. (A) Rectum width (4.5 mm, sagittal T1 image). (B) Sigmoid width (4.7 mm, sagittal T1 image). (C) Splenic flexure width (3.4 mm, coronal T1 image). (D) Hepatic flexure width (9.5 mm, coronal T1 image).

**Figure 2.**
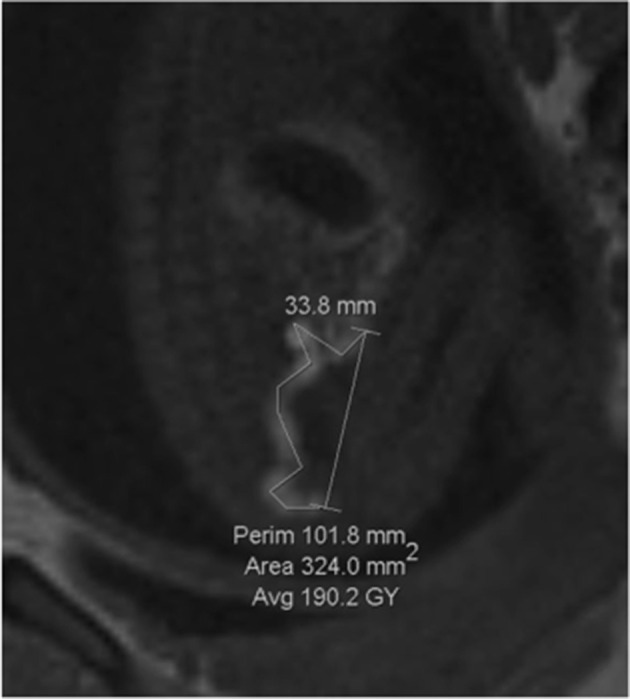
Bowel length measurement using the perimeter and caliper tools. The measurement for the length of the bowel in this image is 68 mm, sagittal T1 image.

Meconium accumulation was studied as a means of evaluating the calibre of the bowel on MRI. The width of the meconium in millimetres (mm) at the widest part of the rectum, sigmoid colon, splenic flexure and hepatic flexure was measured using the proprietary software with the caliper tool (Figure 1a–d). The positions were selected after viewing images in several planes. The length of meconium was measured, from the distal end of the rectum to the most proximal part of the large bowel meconium that could be visualised. The perimeter tool was used to measure the visible length of meconium, but must form a closed shape so the caliper tool was used to measure the excess and this number was then subtracted from the total perimeter to give a measurement for the length (Figure 2). In cases where the length was present in different images in the series, the measuring tool was placed at the end of the previous line when starting the next image, to try and ensure accuracy. If meconium was seen in the small bowel, it was noted in terms of presence or absence but not measured. In all cases, the image in the series that had the best visualisation of meconium was used.

Outliers of each week were identified with the modified z-score method using the median and median absolute deviation (MAD) of the data. Meconium width and length measurements were correlated with gestational age, to the nearest week. Pearson’s correlation coefficients (r values) and *p*-values were calculated for each data set. A *p*-value of < 0.05 was deemed statistically significant throughout. For inter-rater reliability, a representative sample of 10 random scans was remeasured by a trained observer (expert with over 20 years’ experience of foetal MRI) and untrained observer (PhD student with less than a years’ experience of foetal MRI) and compared with original measurements. For intrarater reliability, the same sample was measured again 8 weeks later by the original rater.

## Results

181 foetal body scans were potentially eligible and were examined. 52 were excluded leaving 129 confirmed for analysis. 29 scans were excluded due to insufficient scan quality, 13 due to lack of T1 images or use of a 3 T scanner and 10 due to bowel pathology being detected. Outcome data were collected for all 129 cases. Meconium visibility allowed measurement of length in 128 of the 129 included cases – one case did not have any meconium in the large bowel to measure, and only showed presence of meconium in the small bowel. Rectal meconium width was measured in 125, sigmoid colon in 119, splenic flexure in 80 and hepatic flexure in 52. The age at time of scan ranged from 17 to 39 weeks (mean ± 1 SD of 26.1 ± 5.1 weeks) with most scans performed at 22 weeks gestation (*n* = 20).

Table 1 displays the percentage of cases in which each part of the bowel’s meconium was seen, for each week of gestation. The rectal meconium was visualised as early as 17 weeks and was the most consistently seen throughout gestation. The meconium in the sigmoid colon was first visualised at week 19 and seen consistently from week 21 onwards. The splenic flexure meconium was also visualised from week 21 onwards, with the exception of week 28. The hepatic flexure meconium was visualised from 21 weeks, but was not visualised at weeks 27 or 28. Visualisation of meconium increased throughout gestation. The results also indicate that meconium accumulates from the distal to proximal large bowel.

**Table 1.** Percentage of recognition of meconium at each part of the bowel for the cases at each gestational week

Week of gestation	N	Rectum (%)	Sigmoid (%)	Splenic flexure (%)	Hepatic flexure (%)	Small bowel (%)
17	1	100.00	0.00	0.00	0.00	0.00
18	1	100.00	0.00	0.00	0.00	0.00
19	1	100.00	100.00	0.00	0.00	0.00
20	1	100.00	0.00	0.00	0.00	100.00
21	19	94.74	84.21	36.84	15.79	15.79
22	20	100.00	90.00	25.00	20.00	30.00
23	17	100.00	100.00	47.06	29.41	41.18
24	8	100.00	100.00	75.00	37.50	37.50
25	8	100.00	100.00	87.50	25.00	12.50
26	5	100.00	100.00	100.00	40.00	40.00
27	3	66.67	33.33	33.33	0.00	33.33
28	2	100.00	100.00	0.00	0.00	0.00
29	7	85.71	100.00	85.71	57.14	14.29
30	6	83.33	100.00	83.33	66.67	50.00
31	3	100.00	100.00	100.00	100.00	0.00
32	6	100.00	100.00	100.00	66.67	16.67
33	8	100.00	100.00	100.00	87.50	37.50
34	5	100.00	100.00	100.00	80.00	20.00
35	2	100.00	100.00	100.00	100.00	50.00
36	1	100.00	100.00	100.00	100.00	0.00
37	1	100.00	100.00	100.00	100.00	0.00
38	3	100.00	100.00	100.00	66.67	0.00
39	1	100.00	100.00	100.00	100.00	0.00

Meconium in the small bowel was visualised more intermittently than in the large bowel, and visualisation did not increase linearly with gestational age (Table 1). It was seen as early as 20 weeks but then not visualised in weeks 28, 31 or from week 36 onwards. In 17 cases, meconium was seen in the small bowel and distal large bowel, but not the proximal large bowel.

**Figure 3.**
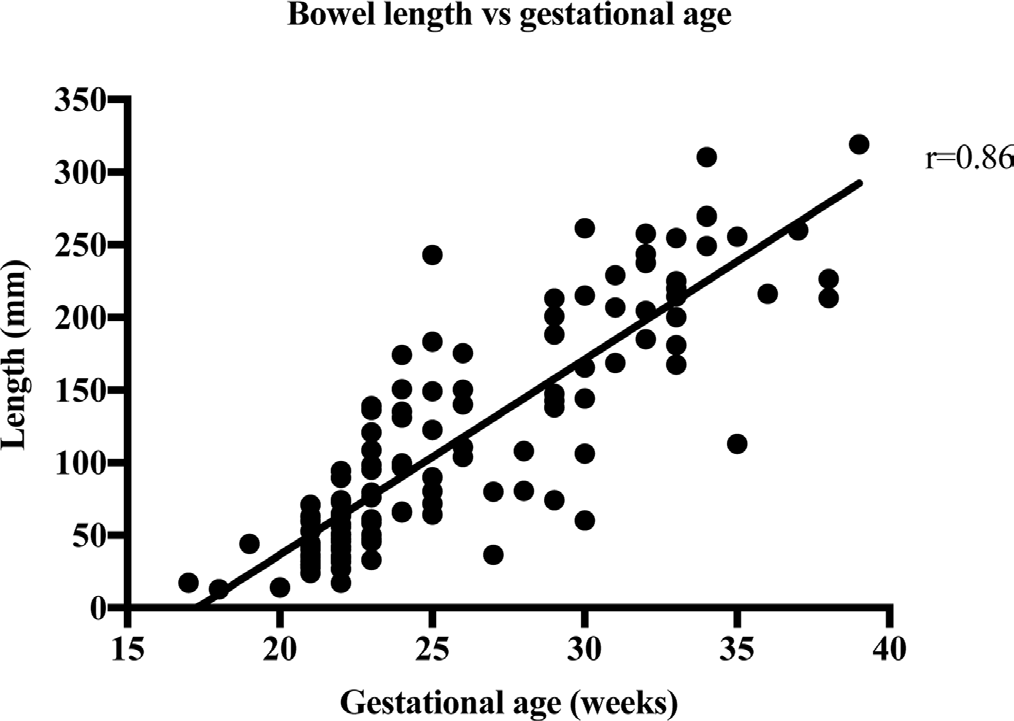
The length measurements of 119 foetal bowels are plotted against the week of gestation.

**Figure 4.**
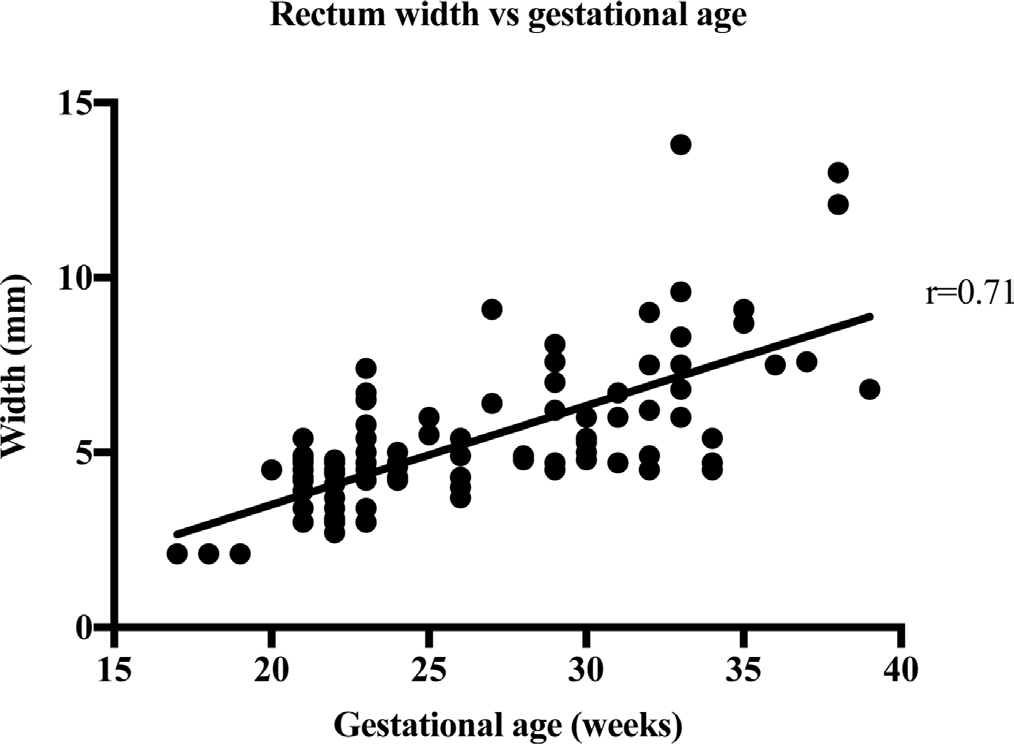
The width measurements of 114 foetal rectums are plotted against the week of gestation.

**Figure 5.**
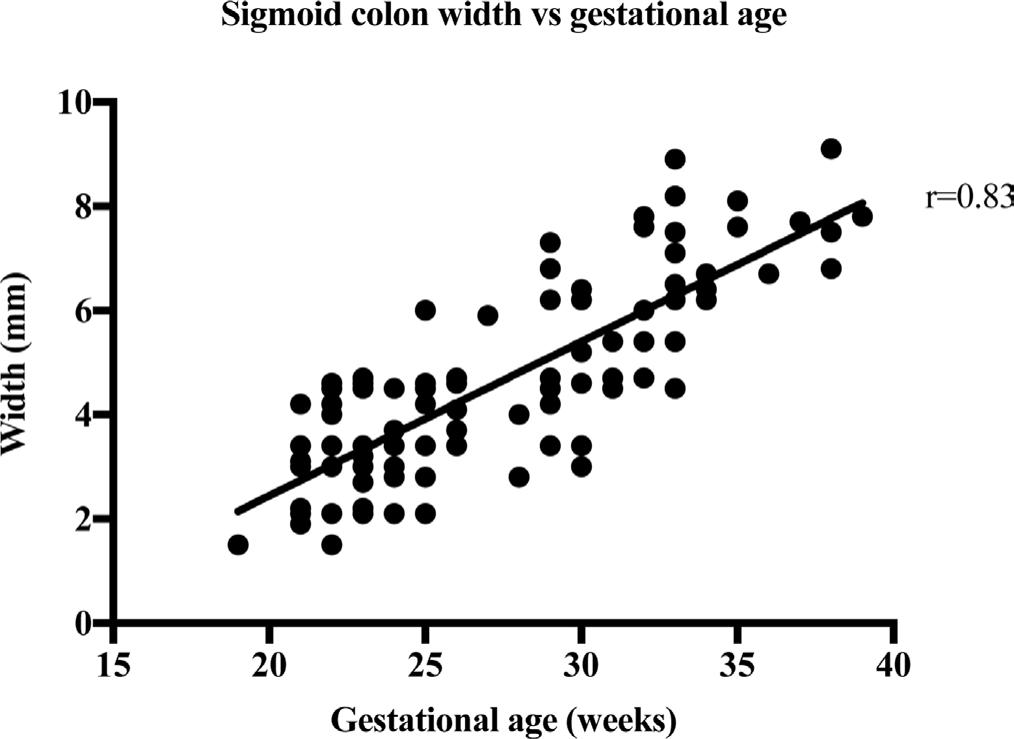
The width measurements of 113 foetal sigmoid colons are plotted against the week of gestation.

**Figure 6.**
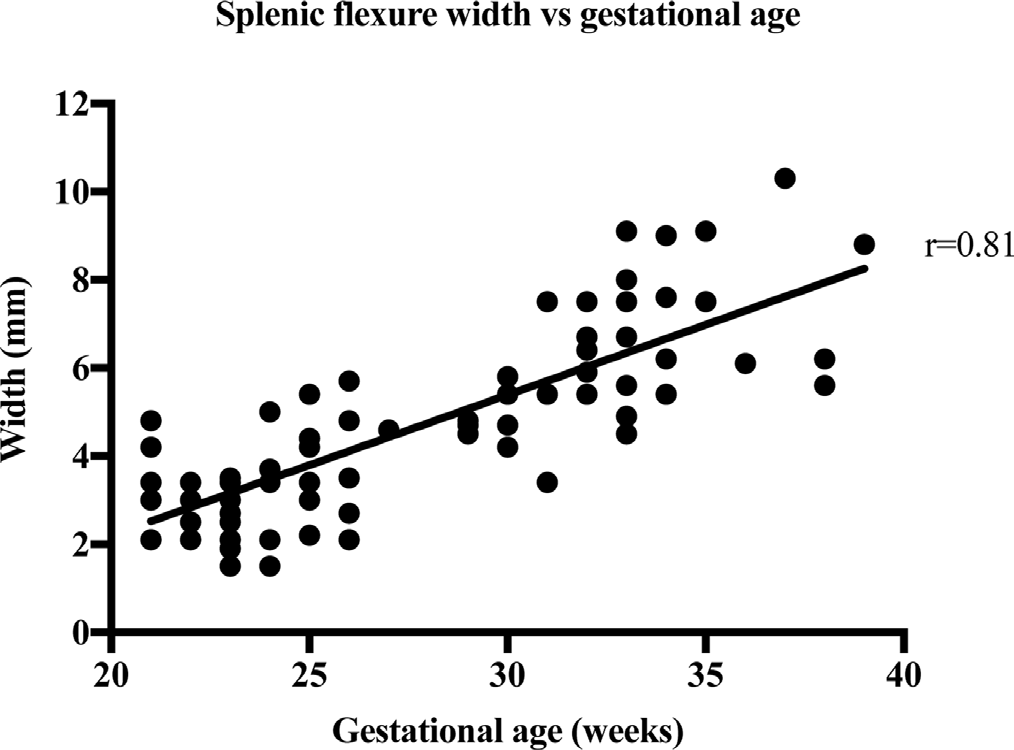
The width measurements of 77 foetal splenic flexures are plotted against the week of gestation.

The strength of the linear relationship between length and width of the bowel meconium and gestational age was established by Pearson’s correlation coefficient, r. Table 2 shows the results of the correlation analysis and the equivalent graphs (Figure 3, Figure 4, Figure 5, Figure 6, ) for each variable. There is a statistically significant (*p* < 0.0001) strong (*r* = 0.6–1.0) or very strong (*r* = 0.8–1.0) positive correlation for all measurements when compared with gestational age. This indicates that the visible length of the meconium increases throughout gestation, and that width also increases with gestational age at each anatomical location.

**Table 2.** Results of statistical analysis for bowel length and widths and corresponding figure number

Part of bowel	N after outlier removal	Range (mm)	Mean ± 1 SD (mm)	r value	r^2^	95% CI	*p*-value	Corresponding figure
Length	119	13.1–319.I	116.8 ± 78.93	0.86	0.73	0.80–0.90	<0.0001	3
Rectum width	114	2.1–13.8	5.20 ± 2.06	0.71	0.50	0.60–0.79	<0.0001	4
Sigmoid colon width	113	1.5–9.1	4.38 ± 1.81	0.83	0.70	0.77–0.88	<0.0001	5
Splenic flexure width	77	1.5–10.3	4.78 ± 2.03	0.81	0.65	0.71–0.87	<0.0001	6
Hepatic flexure width	50	1.5–10.1	5.17 ± 2.24	0.69	0.48	0.51–0.81	<0.0001	7

SD, standard deviation.

**Figure 7.**
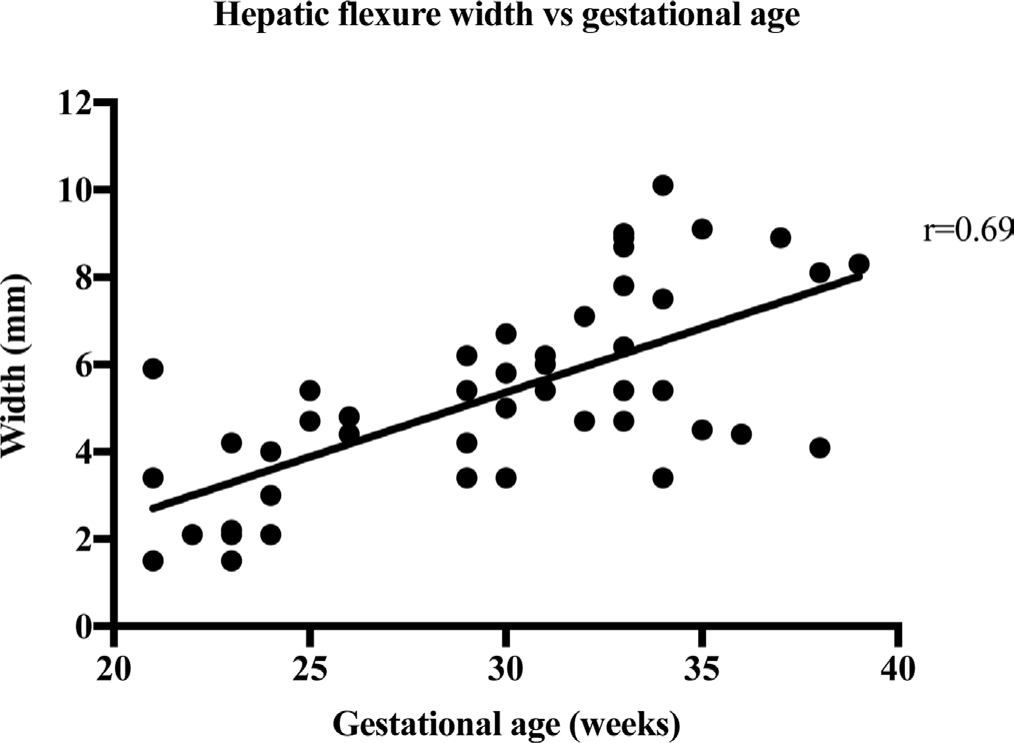
The width measurements of 50 foetal hepatic flexures are plotted against the week of gestation.

Follow-up data were obtained for all cases. Of the 129 cases, only 7 were referred due to potential bowel issues, all of which had normal MR images and were normal at birth before discharge. The other 122 cases were referred predominantly for renal abnormalities, abdominal cysts, liver abnormalities or heart and lung abnormalities. All of these had no bowel problems after delivery. Two cases referred for renal problems and two for spinal problems had post-mortems, with no bowel abnormalities reported.

Both a trained (repeat 1) and untrained (repeat 2) observer, distinct to the original observer, completed 10 random measurements from the results cohort and the measurements were compared to the original values (Table 3). All measurements had moderate to excellent correlation (*r* = 0.4–1.0), with the best correlation seen for the length of meconium and the rectal meconium width measurements and the worst for the sigmoid meconium width. The presence or absence of meconium in the small bowel was also noted; with the original and trained observers identifying it in 2 of the 10 scans. The untrained observer noted meconium was present in 9 of the 10 scans, reflecting the lack of training. The results of the repeated measurements by the original rater can be seen in Table 4. There was good to excellent (0.78–0.97) intrarater reliability throughout. These results indicate that measurements obtained by this method are reliable in experienced observers and reproducible.

**Table 3.** Inter-rater reliability results. (Repeat 1 = trained, Repeat 2 = untrained)

Part of bowel	Rater	Mean (mm)	SD	ICC ( *vs* Rater 1)	95% CI	Strength of correlation
Bowel length (*n* = 10)	Original	109.82	88.40			
	Repeat 1	103.25	88.27	0.96	0.85–0.99	Excellent
	Repeat 2	71.68	53.38	0.77	0.31–0.94	Good
Rectum width (*n* = 10)	Original	4.63	1.36			
	Repeat 1	4.45	1.52	0.78	0.34–0.94	Good
	Repeat 2	5.55	2.00	0.60	−0.01–0.88	Good
Sigmoid colon width (*n* = 7)	Original	4.34	0.91			
	Repeat 1	4.36	0.94	0.43	−0.40–0.97	Moderate
	Repeat 2	5.17	1.38	0.59	−0.21–0.91	Moderate
Splenic flexure width (*n* = 5 original and repeat 1, *n* = 4 repeat 2)	Original	5.36	1.94			
	Repeat 1	5.08	1.33	0.56	−0.47–0.94	Moderate
	Repeat 2	7.2	2.56	0.81	−0.23–0.99	Excellent
Hepatic flexure width (*n* = 5 original and repeat 1, *n* = 4 repeat 2)	Original	4.52	1.55			
	Repeat 1	5.44	2.05	0.55	−0.48–0.94	Moderate
	Repeat 2	5.73	2.37	0.63	−0.56–0.97	Good

ICC, intraclass correlation coefficient; SD, standard deviation.

**Table 4.** Intrarater reliability results

Part of bowel	Measurement	Mean (mm)	SD	ICC ( *vs* original)	95% CI	Strength of correlation
Bowel length (*n* = 10)	Original	109.82	88.40			
	Repeat	102.95	84.72	0.97	0.88–0.99	Excellent
Rectum width (*n* = 10)	Original	4.63	1.36			
	Repeat	4.39	1.57	0.97	0.89–0.99	Excellent
Sigmoid colon width (*n* = 7)	Original	4.34	0.91			
	Repeat	4.37	0.92	0.78	0.24–0.96	Good
Splenic flexure width (*n* = 5)	Original	5.36	1.94			
	Repeat	5.66	2.13	0.93	0.59–0.99	Excellent
Hepatic flexure width (*n* = 5)	Original	4.52	1.55			
	Repeat	4.76	1.58	0.97	0.80–1.00	Excellent

ICC, intraclass correlation coefficient; SD, standard deviation.

## Discussion

The rectal meconium was visualised most consistently from 17 weeks gestation, followed by the sigmoid colon from 19 weeks onwards, and the splenic and hepatic flexures intermittently from 21 weeks and more consistently from 31 and 35 weeks respectively. It can be concluded that in the large bowel meconium accumulates in the rectum first, from as early as 17 weeks. The meconium then accumulates in a distal to proximal direction through the sigmoid colon, splenic flexure and hepatic flexure with gestational age. It has been suggested in the literature that meconium is present in the rectum from 13 weeks,^8^ but this has not been supported by any primary studies. The earliest gestational age we had in this study was 17 weeks and meconium was visualised in the rectum in this case.

Saguintaah et al^8^ previously reported distal to proximal accumulation of meconium in their study of 40 cases providing details in 2 groups, appearances pre-32 weeks or post-32 weeks gestational age and rectal and colonic measurements at each gestational age between 24 and 38 weeks. They found that the meconium in the rectum and sigmoid colon was visualised most effectively, followed by the left colon, transverse colon and right colon, and visualisation increased in all areas with gestation. This is the same pattern of accumulation seen in the present larger study. Inaoka et al^9^ reported similar results in their study of 28 normal cases, with all the studied scans showing high signal intensity on *T*_1_W MRI in the rectum, sigmoid, descending and transverse colon from 20 weeks gestation, and lower signal intensity in the ascending colon, indicating proximal bowel is the last to fill with meconium. The current study confirms these initial observations in a larger cohort and provides additional detail in terms of the width and length of the meconium pattern.

Strong correlation between visible meconium length and gestational age suggests that meconium accumulation increases with increasing gestational age. This may be helpful in determining the presence and type of bowel pathology. A 2009 paper did not measure the length of bowel but found that the volume of the normal colon increases exponentially with gestational age, which would be reflective of the length and width increasing.^11^

Strong correlation between the width of meconium at every part of the bowel and gestational age shows that bowel width also increases throughout gestation. Malas et al^12^ have previously reported on normal colon width increasing with gestational age, especially after the first trimester and this increase in meconium width supports this finding. This is supported by a 2018 paper by Ben-Nun et al^13^ which found that the width the left, right, transverse colon and rectum increased exponentially with gestational age. The width of the rectum varied from 2.1 to 13.8 mm in this study. This agrees with the work by Saguintaah et al^8^ and Ben-Nun et al.^13^ These results are similar to the width of the colon measured on ultrasound.^14^ The correlation of increasing width with increasing gestational age was almost the same (*r* = 0.82 on ultrasound, *r* = 0.83 for sigmoid colon on MRI). The ultrasound data report the colon in general, unlike MRI that can identify the different anatomical areas of the colon. The ultrasound data reported width ranges from 4 to 6 mm at 22 weeks to 10 to 18 mm at term, which is similar to the MRI data reported here ranging from 1.5 mm at 17 weeks to 13.8 mm at 39 weeks.

Additional data are required to generate smooth growth curves: however, when clinically viewing scans, any values markedly larger or smaller than averages for each week of gestation would be considered outside the measured ranges found in this study. Knowledge of the normal pattern of meconium accumulation will be useful to detect conditions where this accumulation is disturbed. This could include raising suspicion for malrotation, ileal atresia, microcolon, and bowel dilatation in cases with gastroschisis or exomphalos. It will also aid in the detection of potential cloacal abnormalities and GI to genitourinary fistulae amongst other pathologies.^7^ Lack of meconium visualisation in the rectum at any gestational age beyond 17 weeks may alert clinicians to potential bowel pathology. These changes are more important in cases where the foetus may have a syndrome with multiple clinical features.

Good inter- and intrarater reliability for the meconium length and width in each location indicates that the method is robust and easily applied; however, presence of meconium in the small bowel was poorly identified by the untrained observer. The small bowel meconium was visualised from week 20, and there was intermittent visualisation until 35 weeks. These findings agree with the literature.^8,13^ At earlier gestations, the meconium in the small bowel would appear bright on T1 scans as it is more concentrated, as the digestive system is less developed so may not move contents through as fast. After 35 weeks, the small bowel meconium was not visualised on *T*_1_W images but was fluid filled and of high signal intensity on *T*_2_W images. This may be due to the foetal swallowing reflex being more developed, resulting in the contents of the small bowel becoming more dilute and fluid filled, and therefore less bright on T1. This is supported by Saguintaah et al^8^ and Inaoka et al^9^ who reported that signal intensity on T1 images of the small bowel was high in earlier gestations, while T2 signal was high in later gestations. Saguintaah et al^8^ also demonstrated a proximal to distal accumulation of fluid in the small bowel, beginning from the jejunum. It has previously been thought that the small bowel is filled with amniotic fluid throughout gestation;^15,16^ however, current evidence does not appear to support this.

In this study, there were 17 cases where meconium was visualised in the rectum, sigmoid colon and small bowel but not in the splenic or hepatic flexure. This does not completely agree with the findings from large bowel analysis that meconium accumulates from the distal to proximal bowel. This is in keeping with the Saguintaah 2002 paper that states that meconium is present in the small bowel but variably,^8^ as it is migrating to the large intestine, and the proximal colon has not yet stored the meconium as it is accumulating from the distal bowel.

The main limiting factor of the study was the uneven spread of gestational age of the cases in the cohort especially at 28 weeks and 35 weeks where there were just two cases, and there was only one case at the beginning and end of our age range. However, this is a large data set providing additional data to the small cohorts already published and increases the confidence in the data when studying cases of foetal gastrointestinal tract abnormality. Additionally, there was measurement imprecision due to the images being acquired with an in-plane resolution of 1.5 mm. This issue will have introduced some error into the measurements and may have had a minor effect on inter- and intrarater reliability correlation. Nonetheless, the values of all raters showed an approximately normal distribution excluding systematic measurement error by each rater. The image sequences used and resolution reflects clinical practice, making the results transferable.

## Conclusions

The findings of this study provide a larger more detailed data set than currently published providing further information concerning the normal accumulation of foetal meconium on MR imaging. Strong correlation of meconium length and width with gestational age was demonstrated in a large cohort. Meconium was found to accumulate from the distal to proximal large bowel throughout gestation. In the small bowel, meconium was seen to be present in earlier gestations and then replaced or diluted with amniotic fluid. This study provides a basic biometric dataset for normal dimensions of the meconium visible in the foetal bowel throughout gestation. With analysis of further cases growth curves can be produced for use in clinical practice similar to those already available for the central nervous system structures to help identify potential pathology and improve patient management.
